# Development of Malate Biosensor-Containing Hydrogels and Living Cell-Based Sensors

**DOI:** 10.3390/ijms252011098

**Published:** 2024-10-16

**Authors:** Nathan J. Ricks, Monica Brachi, Kevin McFadden, Rohit G. Jadhav, Shelley D. Minteer, Ming C. Hammond

**Affiliations:** 1 Department of Chemistry, University of Utah, Salt Lake City, UT 84112, USA; 2 Henry Eyring Center for Cell and Genome Science, University of Utah, Salt Lake City, UT 84112, USA; 3 Kummer Institute Center for Resource Sustainability, Missouri University of Science and Technology, Rolla, MO 65409, USA

**Keywords:** DcuS, Cache, bioelectrode, cell-based biosensor

## Abstract

Malate is a key intermediate in the citric acid cycle, an enzymatic cascade that is central to cellular energy metabolism and that has been applied to make biofuel cells. To enable real-time sensing of malate levels, we have engineered a genetically encoded, protein-based fluorescent biosensor called Malon specifically responsive to malate by performing structure-based mutagenesis of the Cache-binding domain of the Citron GFP-based biosensor. Malon demonstrates high specificity and fluorescence activation in response to malate, and has been applied to monitor enzymatic reactions in vitro. Furthermore, we successfully incorporated Malon into redox polymer hydrogels and bacterial cells, enabling analysis of malate levels in these materials and living systems. These results show the potential for fluorescent biosensors in enzymatic cascade monitoring within biomaterials and present Malon as a novel tool for bioelectronic devices.

## 1. Introduction

Malate is a C4 dicarboxylate that serves as an essential intermediate in the tricarboxylic acid cycle (TCA) [1,2]. There is growing interest in using TCA enzymes in bioelectrodes for energy conversion applications. Enzymatic biofuel cells involve multi-enzymatic cascades able to carry out the sequential bio-electrochemical oxidation of the substrates. Ongoing research focuses on improving the efficiencies of these biofuel cells to collect a higher number of electrons and reach higher power densities [3,4,5,6].

Enzymatic cascades fabricated with TCA cycle enzymes can be potentially used for achieving a high degree of oxidation of substrates, including ethanol, pyruvate, and lactate [7,8]. One strategy to improve the efficiencies of these multi-enzymatic cascades is to improve the transfer of intermediates from one enzyme active site to another [5,9]. In this context, there is a need for sensitive and selective sensors that can visualize and monitor the intermediates generated in situ by these enzymatic cascades in the functional material. Specifically, sensing malate could enable the further development and optimization of bioelectrodes for energy conversion, because this intermediate is known to build up due to inefficient conversion by malate dehydrogenase in the enzymatic cascade.

Several previous platforms have been developed for sensing malate, including two genetic reporter assays based on two-component systems, one in *E. coli*, the other in *B. licheniformis* [10,11]. Additionally, a malate sensor was designed using a malate-responsive protein domain labeled with the dansyl fluorophore [12]. However, these previous approaches have limitations, as they do not function both in vitro and in vivo, the reporter has slow response time, and the dansylated biosensor requires external chemical labeling. Here, we aim to develop a genetically encoded, protein-based fluorescent malate sensor that can be used for both in vitro and in vivo applications, has faster response time, and is label free. The dual application was of interest because we considered that sensors could be embedded in a functional material such as a hydrogel either as the protein biosensor itself or as a living cell-based sensor.

Many fluorescent biosensors have been developed through the use of fluorescent proteins (FPs) such as GFP [13,14,15]. Linking FPs to protein sensing domains can result in a functional biosensor when analyte binding to the sensing domain propagates a shift in conformation that changes the physiochemical environment of the FP chromophore and modulates fluorescence [15]. An extracellular sensing domain commonly found in bacteria are Cache domains, which are often linked to a catalytic histidine kinase, methyl chemotaxis, or the GGDEF domain. These sensing domains have been shown to respond to a variety of extracellular ligands, ranging from amino acids, to dicarboxylic acids, to quorum signals [16,17,18]. When the cognate ligand binds to the Cache-sensing domain, a conformational change is propagated across the transmembrane helix to modulate activity of the catalytic domain. While various models have been proposed for the mechanism of conformational change, including the piston, scissor blade, and rotating helix models [19,20,21], all models attribute the conformational change propagating across the transmembrane helix. The inherent conformational change upon ligand binding makes Cache domains attractive targets for designing FP-based biosensors, with a recent precedent being the development of Citron, a biosensor that employs a citrate-responsive Cache [13]. Inspired by Citron, we have developed an FP-based biosensor selective for malate called Malon, which was successfully incorporated into bacterial cells and hydrogels, enabling analysis of malate levels in these systems.

## 2. Results

### 2.1. Design of Malon Based on Domain Replacement and Pocket-Grafting Strategies

**Figure 1:**
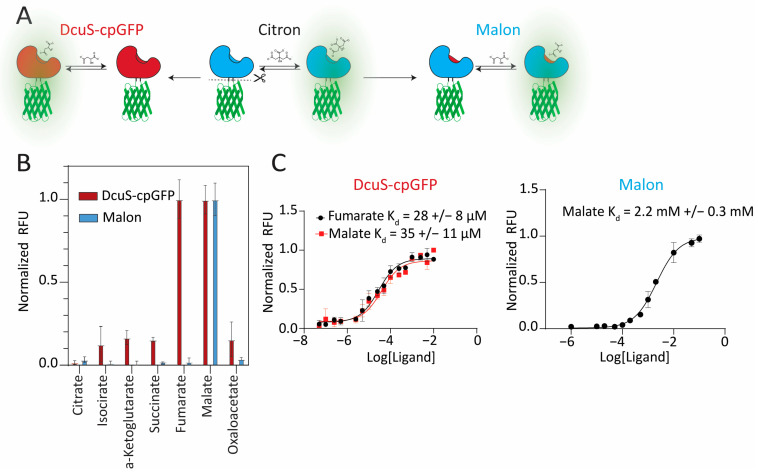
Design and characterization of DcuS–cpGFP and Malon biosensors for malate. (**A**) The malate responsive Cache domain, DcuS, was linked to GFP to generate DcuS–cpGFP. Alternatively, the DcuS-binding pocket was grafted to the previously engineered Citron biosensor to generate Malon. (**B**) Normalized fluorescence response in vitro for biosensors to 100 mM of malate or other Krebs cycle intermediates. (**C**) Fluorescence binding assays to determine binding affinities.

In our first approach to constructing a malate biosensor, the citrate-sensing Cache domain from the Citron biosensor (Addgene plasmid #134300) [13], which was derived from the *Klebsiella pneumoniae* CitA gene, was replaced with residues 30–159 of the Cache domain of *E. coli* DcuS based on sequence and structural homology (Figure 1A, Figures S1 and S2). Prior in vivo and crystallographic data supported that DcuS responds to malate [10,22]. DcuS was inserted at the same location within β-strand 7 of circularly permuted (cp) GFP as CitA, between the gate post residues flanking a two-residue bulge, a site known to tolerate insertions well [13,15].

Replacement of CitA with DcuS initially resulted in a nonfluorescent protein construct, which indicates that the linker sequences were sub-optimal. Thus, each two amino acid long linker, in turn, was randomized by saturation mutagenesis, and a small library (~200) of randomized constructs was screened for fluorescence recovery by flow cytometry, followed by assaying fluorescent candidates for response to malate in cell lysates. The variant with the greatest increase in fluorescence upon malate addition was found to have a ΔRFU/RFU of 0.5 in cell lysate and was designated as DcuS–cpGFP. The purified biosensor exhibited a higher ΔRFU/RFU value of ~1.8, most likely due to malate in cell lysates leading to a background signal.

DcuS–cpGFP had a good binding affinity to malate (K_D_ ~ 35 μM). However, it responded similarly to fumarate (K_D_ ~ 28 μM) (Figure 1B,C). To check whether the selectivity was being affected by the GFP or linker portions of the biosensor, we evaluated ligand binding to DcuS alone using a thermal shift assay, which showed that both malate and fumarate bind to this Cache domain (Figure S3). After further investigation, we found that there were prior experimental data supporting both malate and fumarate as strong effectors of conformational change in DcuS [22].

Concurrent with the development of DcuS–cpGFP, a second approach was pursued from a structure-based design perspective. Due to extensive homology between DcuS and CitA (Figure S2), we hypothesized that grafting the binding pocket of DcuS into the Citron sensor would result in a biosensor that would bind malate. This hypothesis was supported by a previous study in which the *Geobacillus thermoleovarans* CitA Cache domain was converted into a malate sensor through binding pocket mutagenesis based on DcuS and conjugation of fluorescent dyes [12]. Additionally, a previous study demonstrated the similarity of the CitA- and DcuS-binding pockets by converting DcuS to have citrate specificity by mutating the pocket to increase the size to accommodate citrate [23].

Five mutations were made in the binding pocket of the Citron sensor (G204T, M223F, S228I, K253F, S268A), which resulted in Malon (Figure 1A). The purified Malon biosensor exhibited a ΔRFU/RFU value of ~10 in response to malate addition, which was significantly higher than DcuS–cpGFP and was expected with the extent of linker optimization and directed evolution previously performed to make Citron (reported ΔRFU/RFU of ~9) [13]. Interestingly, despite the mutations being based on the DcuS-binding pocket, which we showed is promiscuous for malate and fumarate, Malon was selective for malate over all other TCA compounds, including citrate and fumarate (Figure 1B and Figure S4). However, the grafted binding pocket reduced affinity to malate (K_D_ ~ 2.2 mM, Figure 1C) relative to the native Cache domain from DcuS, which was expected, as the Citron biosensor had a similar affinity (K_D_ ~ 1.1 mM).

The fluorescent properties of the two new biosensors, Malon and DcuS–cpGFP, were analyzed and compared to those reported for Citron (Table 1). The excitation and emission profiles for the three biosensors were nearly identical (Figure S5). The addition of ligand had a larger effect on the extinction coefficient than quantum yield for these biosensors, but both values did increase, which led to the increased brightness and observed fluorescence activation.

GFP-based sensors function by altering the chromophore’s pK_a_ upon analyte binding, causing a shift between a dim phenol form and a more fluorescent phenolate form. However, this mechanism can cause artifactual increases in fluorescence due to pH changes rather than analyte binding [15]. Therefore, we measured Malon’s fluorescence as a function of pH ranging from 4 to 11 (Figure S6). The ΔRFU/RFU value was calculated as the increase in fluorescence upon malate addition divided by the background fluorescence, which served as a metric for the signal change of the sensor. Overall, the sensor was functional across the full range of pH values except pH 4, but the fluorescence signal change was highest for pH 5–6, whereas it remained consistent for pH 7–11. Subsequent in vitro experiments were run near physiological pH at 7.5.

Cellular malate concentrations have been reported to be in the low millimolar range. In *E. coli*, malate concentrations were reported to be between 0.9 and 1.5 mM, while in eukaryotic cells, malate was found to be between 0.2 and 0.5 mM [24]. Furthermore, catalytic biomaterials based on TCA enzymes for energy-harvesting applications are often analyzed using substrates in millimolar concentrations. Thus, we expect that Malon would be a useful biosensor, due to its high selectivity, high signal turn-on, and appropriate range of sensitivity for malate.

### 2.2. Investigation of Ligand Recognition in the Binding Pocket

Our results demonstrate that even though DcuS and CitA are highly structurally homologous Cache domains, their ligand-binding pockets either accept or discriminate against fumarate, respectively. Using the DcuS x-ray crystal structure with bound malate (PDB code 3by8), malate was removed and fumarate was docked in using Autodock Vina. Residues within 10 Å of the original malate-binding site were treated as flexible side chains and allowed to move to accommodate fumarate. The docked model was then compared to the native structure with bound malate. The highest-scoring docked model showed fumarate bound to the DcuS pocket in a comparable position to malate, but the double bond in fumarate changed the orientation of the carboxylate that normally forms a salt bridge with R211 (Figure S7B). The docked model shows that, to accommodate fumarate, the carboxylate interacts with both R211 and R219. In contrast, the homologous positions in the CitA-binding pocket were residues R210 and E218.

Based on this ligand-docking model, we hypothesized that R219 is responsible for the promiscuity of DcuS for fumarate. Indeed, the R219E mutant of DcuS–cpGFP gained selectivity for malate over fumarate (Figure S7F). Interestingly, however, the E218R mutant of Malon remained selective, even though we would expect this reverse case to become promiscuous for fumarate. Both mutants exhibited decreased binding affinity to malate (Figure S7E,F). Taken together, these results suggest that R219 may play a necessary but not sufficient role in fumarate binding.

Malon was developed by grafting the primary coordination sphere of DcuS into the CitA-binding pocket. To try to improve the binding affinity to malate, we made structure-based mutations of residues in the secondary coordination sphere. However, none of these additional mutants improved the binding affinity in a significant manner (Figure S8).

### 2.3. Monitoring and Quantitating Malate in Enzymatic Assays

Developing effective biomaterial and biocatalytic devices often involves enzyme modifications, such as immobilization, mutagenesis, or enzymatic fusions [25,26,27]. These strategies, while necessary for application to devices, can negatively impact enzymatic activity. Consequently, rapid and reliable assessment of enzyme activity in modified enzymes is important for optimizing and validating enzyme formulations.

**Figure 2:**
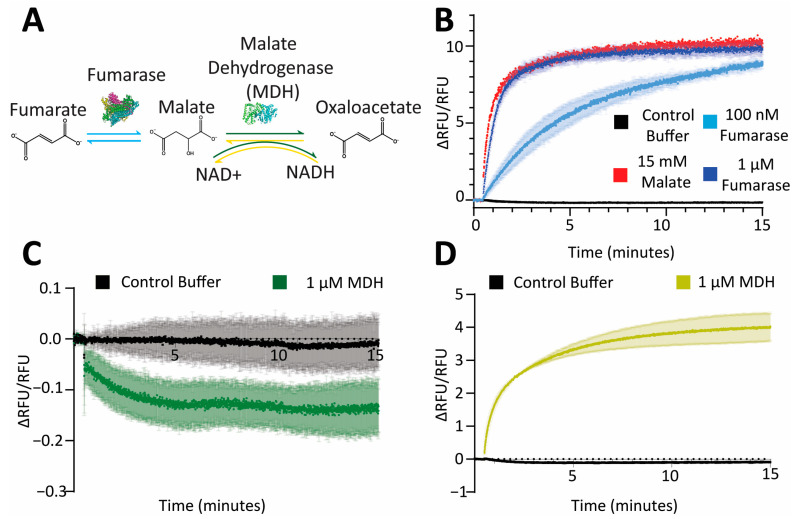
In vitro enzyme assays were analyzed with Malon. (**A**) Schematic of the enzyme reactions being assessed. Fumarase converts fumarate to malate, and malate dehydrogenase (MDH) oxidizes malate to oxaloacetate by reducing NAD^+^ to NADH, or vice versa. (**B**) Malon fluorescence in enzyme reactions with fumarate and 0, 100 nM, or 1 μM fumarase, compared to direct detection of 15 mM malate. (**C**) Malon fluorescence in enzyme reactions with 0 or 1 μM MDH pre-equilibrated with malate upon NAD^+^ injection. (**D**) Same as part C, except pre-equilibrated with oxaloacetate upon NADH injection.

To demonstrate the applicability of Malon in a 96-well format assay, in vitro experiments were performed on two Krebs cycle enzymes, fumarase that produces malate and malate dehydrogenase (MDH) that consumes malate in the forward direction (Figure 2A). Injection of fumarate substrate to wells containing fumarase and Malon yielded an expected increase in fluorescence over time as malate was produced (Figure 2B). Conversely, injection of NAD^+^ into wells containing MDH, malate, and Malon led to a decrease in fluorescence over time as malate was consumed to produce oxaloacetate, although the observed change was small because the reaction equilibrium lay far to the left (Figure 2C). The reverse reaction for MDH also can be monitored, as injection of oxaloacetate into wells containing MDH, NADH, and Malon led to a fluorescence increase as malate was produced (Figure 2D).

The maximum enzyme catalytic rate that can be accurately measured depends on the biosensor response rate, which was determined by direct addition of 15 mM malate based on the equilibrium constant for fumarase (4.4) [28]. As shown, the enzymatic reaction with 1 μM fumarase and 18 mM fumarate approached the maximum measurable rate under these conditions (Figure 2B). In addition, the change in fluorescence signal (ΔRFU/RFU) can be converted to malate concentrations by preparing a standard curve for Malon biosensor response with different concentrations of malate (0.75–25 mM) in the linear region of the K_D_ curve (Figure S9A). Using the reported equilibrium constant for fumarase, an expected concentration of malate should form from known concentrations of fumarate substrate. Thus, we compared the malate concentrations from fumarase reactions measured experimentally using the Malon biosensor to the expected malate concentrations based on the equilibrium constant, and found a Pearson correlation coefficient of 0.97 and a root mean square error of 0.63. These data indicate strong agreement between the predicted malate concentrations from the equilibrium constant and those measured using the Malon sensor. Applying this analysis to the in vitro enzymatic reaction with fumarase shown in Figure 2B, the final malate concentration was measured to be 13 ± 2 mM. In bioelectrochemical devices (i.e., biofuel cell or biobattery), accurate measurement of malate concentrations will be important for evaluating reaction progress and rates.

### 2.4. Incorporating Malon to Make Sensor-Containing Hydrogels and Living Cell-Based Sensors

**Figure 3:**
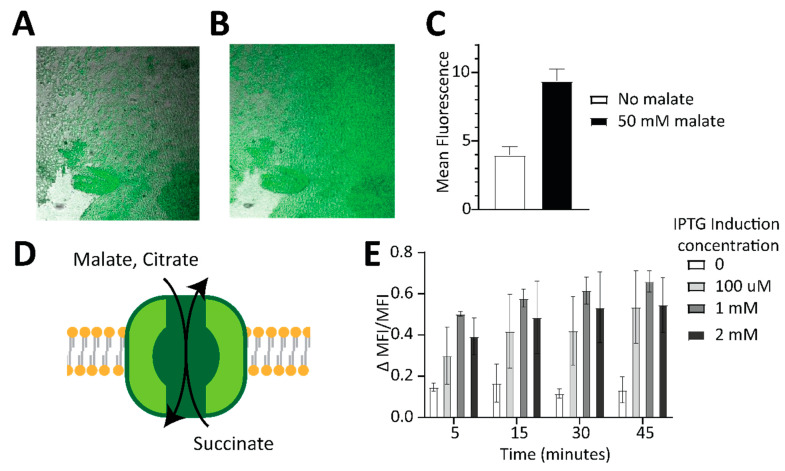
Application of Malon to generate malate-sensing hydrogels and living cells. (**A**,**B**) Transparent ITO electrodes were coated with the crosslinked NQ-LPEI hydrogel polymer premixed with the Malon sensor and placed in a transparent well immersed in phosphate buffer solution pH 7.5 and visualized with a fluorescence confocal microscope before (**A**) and after (**B**) the addition of 50 mM malate. (**C**) Quantitation of mean fluorescence on electrodes before and after malate addition (also see Figure S8). (**D**) The *E. coli* CitT transporter is an antiporter that transports malate and other C4 dicarboxylic acids into the cell. (**E**) Cellular fluorescence measured by flow cytometry of *E. coli* cells expressing Malon and induced with varying amounts of IPTG for CitT overexpression in the presence of 20 mM malate. Shown are data for three biological replicates with standard deviation.

Immobilization of enzymes on electrodes within a hydrogel polymer is a common technique used in bioelectrocatalytic devices, offering advantages such as good biocompatibility and retention of enzyme activity while allowing diffusion of substrates and intermediates, and facilitating electron transfer through redox molecules grafted to the main polymeric backbone [29]. Since oxidoreductases of the TCA cycle require NAD(P) cofactor for their catalytic activity, an electrochemical cofactor regeneration system is the mediation scheme for transferring electrons from the enzymes to the electrode. A linear polymer of ethyleneimine derivatized with naphthoquinone redox pendant groups (NQ-LPEI) is able to mediate the regeneration of the NAD cofactor via self-exchange-based conduction and is a great candidate for enzyme entrapment for future bioelectrocatalytic applications solving the dual role of enzyme immobilization and mediation of cofactor regeneration. However, the activity of enzymes and intermediate species that are not redox active is difficult to study in these systems. To investigate whether the Malon sensor could function embedded in biocatalytic hydrogels, an NQ-LPEI hydrogel polymer crosslinked by ethylene glycol diglycidyl ether (EDGDE) and containing the entrapped Malon sensor was prepared and cast on a translucent indium tin oxide (ITO) electrode, which was used as a transport support. Confocal microscopy was carried out to observe the fluorescence of the sensor inside of the hydrogel (Figure 3A and Figure S10). Upon malate addition to the solution above the hydrogel, a notable increase in fluorescence was observed as the malate diffused inside of the hydrogel to reach the sensor (Figure 3B,C), demonstrating that incorporation of Malon produced a hydrogel that can sense malate directly. While there was high background fluorescence observed in the hydrogel, we attribute this to the high concentration of Malon that was incorporated, because Malon had a similar background to Citron (Figure S5D), and there was no fluorescence signal from the hydrogel alone (Figure S10C). In addition to mixing the sensor at lower concentrations with the polymerization reagents to reduce the background, another workaround we have previously employed is to append a second fluorescent protein to provide a normalization signal [30].

Concurrent with the hydrogel experiments, we also explored making malate-responsive living cells that could be incorporated into bioelectronic devices. However, we found that efficient uptake and detection of malate by bacterial cells required co-expression of CitT, a known transporter of di/tricarboxylic acids such as malate (Figure 3D) [31]. For these experiments, Malon expression in *E. coli* BL21 cells was held constant by arabinose induction, while CitT expression was varied by IPTG concentration and malate was added to the media. Without the CitT transporter, little to no change in fluorescence was observed upon addition of malate. However, increasing expression of CitT led to corresponding increases in ∆MFI/MFI values (Figure 3E), which quantitated the difference in fluorescence between cells with and without malate treatment. These results demonstrate that we created malate-sensing bacterial cells by introducing Malon and CitT. While direct incorporation of Malon into hydrogels likely is preferred over this approach, Malon can be expressed in bacterial cells and applied to measure changes in cellular malate levels as well as assess transporter function.

## 3. Discussion

In this study, we designed GFP-based biosensors for malate with varying levels of sensitivity and specificity by integrating the DcuS Cache domain or grafting the DcuS binding pocket into an existing Cache domain-based sensor. We found that a common insertion site in GFP accommodated both CitA and DcuS Cache domains to generate sensors capable of propagating the conformational change from the Cache-sensing domain to the GFP chromophore environment, enabling ligand binding to modulate fluorescence. While some Cache domains have been characterized, the large sequence space of Cache domains found across genomes remains an untapped source of highly evolved and responsive ligand-binding domains. In 2024, the pfam database classified ~250,000 proteins to harbor a Cache domain in both prokaryotic and eukaryotic genomes [32]. Our work highlights the exciting potential of Cache domains in fluorescent biosensor design.

The performance of the Malon sensor represents an improvement over previously reported sensors. The malate-responsive protein labeled with a dansyl fluorophore had a similar malate-binding affinity to Malon but displayed a modest decrease in signal of ~29% in response to malate, compared to the 1000% signal increase for Malon, which has a similar fluorescence profile to Citron (Figure S5D). Additionally, chemical labeling of a binding protein limits applications to in vitro experiments [12]. Genetic reporters made in *E. coli* and *B. licheniformis* can detect lower malate concentrations than Malon but exhibit much slower response times, ranging from 8 to 24 h [10,11], compared to minutes for Malon (Figure 2B). Genetic reporters also limit applications to the specific bacteria species. In contrast, Malon provides a label-free genetically encodable sensor with a quick response time, large signal change, and broad applicability.

In particular, we showed the application of assessing in vivo enzymatic activity using the Malon sensor. Co-expressing the sensor in *E. coli* with the CitT transporter yielded a living cell-based sensor. While many fluorescence-based biosensors have been successfully developed in bacteria and later employed in eukaryotic cells to monitor cellular dynamics, their application in microbial systems, particularly in the context of metabolic engineering, remains relatively unexplored [14,33]. Recently, interest has grown in engineering probiotic microbes to confer specific health benefits [34]. From metabolic regulation to disease diagnosis, the development of these enhanced probiotics requires metabolic engineering and synthetic biology efforts to create strains with tailored functionalities [35,36,37]. Real-time monitoring through biosensor-based approaches of metabolic states and pathways, such as malate and the TCA, could accelerate the development of probiotics with enhanced therapeutic potential.

Given the extensive testing of the parent sensor, Citron, in eukaryotic cells and its proven functionality, it is expected that Malon also would perform effectively in eukaryotic cells [13]. Further, numerous GFP-based biosensors have been successfully adapted from bacterial to eukaryotic systems while retaining their function [14,33,38]. This work provides a foundation for future studies in eukaryotic systems that could expand the utility of Malon in metabolic research.

Finally, we showed the application of the Malon sensor in assessing enzymatic activity. Co-incubating our sensor with enzymes that consume or produce malate allowed us to visualize the activities of those enzymes in real time. With the growing interest in using TCA enzymes for biomaterials for electrocatalysis applications, novel tools and techniques are needed to assess the activities of the designed systems. In particular, the visualization of the diffusion of the intermediates from the enzymatic catalytic active sites is a fundamental point to test and improve future strategies of substrate channeling of the enzymatic cascade designed to assure a rapid consumption of substrate and intermediates to reach higher product yields and higher electron recoveries from biodevices. Determination of intermediates or products by LC/MS is a common approach but is slow, laborious, and an end-point measurement. Fluorescent sensors such as Malon are a high-throughput method that can provide a fast and reliable response, allowing a direct in situ visualization of enzymatic activity.

In conclusion, we developed Malon, a GFP-based biosensor that rapidly and selectively senses malate in enzymatic assays in vitro and transporter assays in *E. coli*. We provide an initial demonstration that the incorporation of Malon into a redox polymer hydrogel on an electrode surface yields a malate-sensing hydrogel, opening the door to future assessment of the catalytic functions of enzymes in their electrode immobilization matrix. Thus, this work takes the first steps toward the development of in operando measurements of bioelectronic device activity.

## 4. Materials and Methods

Materials and methods used in this study are detailed in Supplementary Materials [39,40,41].

## Figures and Tables

**Table 1:** Characterization of DcuS–cpGFP and Malon compared to the Citron biosensor.

Fluorescent Sensor	Ligand	Extinction Coefficient (mM^−1^ cm^−1^)	Quantum Yield	Brightness	Excitation and Emission Maximum	K_d_	F/F_min_
Citron [13]	+citrate	61.6	0.34	21	499/517	1.1 mM	9
	−citrate	7.2	0.29	0.21	499/516		
DcuS–cpGFP	+malate	26 ± 6	0.28 ± 0.02	7.3	493/515	35 ± 11 μM	1.8 ± 0.3
	−malate	13 ± 4	0.25 ± 0.01	3.3	499/516		
Malon	+malate	110 ± 10	0.32 ± 0.01	35	499/518	2.2 ± 0.3 mM	10 ± 1.2
	−malate	18 ± 5	0.30 ± 0.01	5.4	496/515		

## Data Availability

The original contributions presented in the study are included in the article/Supplementary Material, further inquiries can be directed to the corresponding authors.

## Supplementary Materials

The following supporting information can be downloaded at: https://www.mdpi.com/article/10.3390/ijms252011098/s1.

## References

[1] Krebs H.A. (1940). The Citric Acid Cycle: A Reply to the Criticisms of FL Breusch and of J. Thomas. Biochem. J..

[2] Akram M. (2014). Citric Acid Cycle and Role of Its Intermediates in Metabolism. Cell Biochem. Biophys..

[3] Liu F., Banta S., Chen W. (2013). Functional Assembly of a Multi-Enzyme Methanol Oxidation Cascade on a Surface-Displayed Trifunctional Scaffold for Enhanced NADH Production. Chem. Commun..

[4] Sokic-Lazic D., Minteer S.D. (2009). Pyruvate/Air Enzymatic Biofuel Cell Capable of Complete Oxidation. Electrochem. Solid-State Lett..

[5] Wheeldon I., Minteer S.D., Banta S., Barton S.C., Atanassov P., Sigman M. (2016). Substrate Channelling as an Approach to Cascade Reactions. Nat. Chem..

[6] Xiao X., Xia H., Wu R., Bai L., Yan L., Magner E., Cosnier S., Lojou E., Zhu Z., Liu A. (2019). Tackling the Challenges of Enzymatic (Bio) Fuel Cells. Chem. Rev..

[7] Franco J.H., De Andrade A.R. (2022). Bioelectrodes with Enzyme Cascade Reactions. Advances in Bioelectrochemistry Volume 5: Emerging Techniques and Materials, Biodevice Design and Reactions.

[8] Sokic-Lazic D., Minteer S.D. (2008). Citric Acid Cycle Biomimic on a Carbon Electrode. Biosens. Bioelectron..

[9] Fan S., Liang B., Xiao X., Bai L., Tang X., Lojou E., Cosnier S., Liu A. (2020). Controllable Display of Sequential Enzymes on Yeast Surface with Enhanced Biocatalytic Activity toward Efficient Enzymatic Biofuel Cells. J. Am. Chem. Soc..

[10] Ganesh I., Ravikumar S., Yoo I.-K., Hong S.H. (2015). Construction of Malate-Sensing Escherichia Coli by Introduction of a Novel Chimeric Two-Component System. Bioprocess Biosyst. Eng..

[11] Zhang Y., Li Y., Xiao F., Wang H., Zhang L., Ding Z., Xu S., Gu Z., Shi G. (2021). Engineering of a Biosensor in Response to Malate in Bacillus Licheniformis. ACS Synth. Biol..

[12] Cormann K.U., Baumgart M., Bott M. (2018). Structure-Based Design of Versatile Biosensors for Small Molecules Based on the PAS Domain of a Thermophilic Histidine Kinase. ACS Synth. Biol..

[13] Zhao Y., Shen Y., Wen Y., Campbell R.E. (2020). High-Performance Intensiometric Direct- and Inverse-Response Genetically Encoded Biosensors for Citrate. ACS Cent. Sci..

[14] Nasu Y., Murphy-Royal C., Wen Y., Haidey J.N., Molina R.S., Aggarwal A., Zhang S., Kamijo Y., Paquet M.E., Podgorski K. (2021). A Genetically Encoded Fluorescent Biosensor for Extracellular L-Lactate. Nat. Commun..

[15] Nasu Y., Shen Y., Kramer L., Campbell R.E. (2021). Structure- and Mechanism-Guided Design of Single Fluorescent Protein-Based Biosensors. Nat. Chem. Biol..

[16] Zhang L., Li S., Liu X., Wang Z., Jiang M., Wang R., Xie L., Liu Q., Xie X., Shang D. (2020). Sensing of Autoinducer-2 by Functionally Distinct Receptors in Prokaryotes. Nat. Commun..

[17] Cheung J., Hendrickson W.A. (2008). Crystal Structures of C4-Dicarboxylate Ligand Complexes with Sensor Domains of Histidine Kinases DcuS and DctB. J. Biol. Chem..

[18] Gavira J.A., Gumerov V.M., Rico-Jiménez M., Petukh M., Upadhyay A.A., Ortega A., Matilla M.A., Zhulin I.B., Krell T. (2020). How Bacterial Chemoreceptors Evolve Novel Ligand Specificities. Mbio.

[19] Upadhyay A.A., Fleetwood A.D., Adebali O., Finn R.D., Zhulin I.B. (2016). Cache Domains That Are Homologous to, but Different from PAS Domains Comprise the Largest Superfamily of Extracellular Sensors in Prokaryotes. PLoS Comput. Biol..

[20] Gushchin I., Gordeliy V. (2018). Transmembrane Signal Transduction in Two-component Systems: Piston, Scissoring, or Helical Rotation?. Bioessays.

[21] Salvi M., Schomburg B., Giller K., Graf S., Unden G., Becker S., Lange A., Griesinger C. (2017). Sensory Domain Contraction in Histidine Kinase CitA Triggers Transmembrane Signaling in the Membrane-Bound Sensor. Proc. Natl. Acad. Sci. USA.

[22] Monzel C., Unden G. (2015). Transmembrane Signaling in the Sensor Kinase DcuS of *Escherichia coli*: A Long-Range Piston-Type Displacement of Transmembrane Helix 2. Proc. Natl. Acad. Sci. USA.

[23] Kramer J., Fischer J.D., Zientz E., Vijayan V., Griesinger C., Lupas A., Unden G. (2007). Citrate Sensing by the C4-Dicarboxylate/Citrate Sensor Kinase DcuS of *Escherichia coli*: Binding Site and Conversion of DcuS to a C4-Dicarboxylate-or Citrate-Specific Sensor. J. Bacteriol..

[24] Albe K.R., Butler M.H., Wright B.E. (1990). Cellular Concentrations of Enzymes and Their Substrates. J. Theor. Biol..

[25] Sheldon R.A., van Pelt S. (2013). Enzyme Immobilisation in Biocatalysis: Why, What and How. Chem. Soc. Rev..

[26] Bommarius A.S., Paye M.F. (2013). Stabilizing Biocatalysts. Chem. Soc. Rev..

[27] Kummer M.J., Lee Y.S., Yuan M., Alkotaini B., Zhao J., Blumenthal E., Minteer S.D. (2021). Substrate Channeling by a Rationally Designed Fusion Protein in a Biocatalytic Cascade. J. Am. Chem. Soc..

[28] Krebs H.A. (1953). The Equilibrium Constants of the Fumarase and Aconitase Systems. Biochem. J..

[29] Milton R.D., Wang T., Knoche K.L., Minteer S.D. (2016). Tailoring Biointerfaces for Electrocatalysis. Langmuir.

[30] Dippel A.B., Anderson W.A., Evans R.S., Deutsch S., Hammond M.C. (2018). Chemiluminescent Biosensors for Detection of Second Messenger Cyclic Di-GMP. ACS Chem. Biol..

[31] Pos K.M., Dimroth P., Bott M. (1998). The Escherichia Coli Citrate Carrier CitT: A Member of a Novel Eubacterial Transporter Family Related to the 2-Oxoglutarate/Malate Translocator from Spinach Chloroplasts. J. Bacteriol..

[32] Mistry J., Chuguransky S., Williams L., Qureshi M., Salazar G.A., Sonnhammer E.L.L., Tosatto S.C.E., Paladin L., Raj S., Richardson L.J. (2021). Pfam: The Protein Families Database in 2021. Nucleic Acids Res..

[33] Wu J., Abdelfattah A.S., Zhou H., Ruangkittisakul A., Qian Y., Ballanyi K., Campbell R.E. (2018). Genetically Encoded Glutamate Indicators with Altered Color and Topology. ACS Chem. Biol..

[34] Zhou Z., Chen X., Sheng H., Shen X., Sun X., Yan Y., Wang J., Yuan Q. (2020). Engineering Probiotics as Living Diagnostics and Therapeutics for Improving Human Health. Microb. Cell Factories.

[35] Isabella V.M., Ha B.N., Castillo M.J., Lubkowicz D.J., Rowe S.E., Millet Y.A., Anderson C.L., Li N., Fisher A.B., West K.A. (2018). Development of a Synthetic Live Bacterial Therapeutic for the Human Metabolic Disease Phenylketonuria. Nat. Biotechnol..

[36] Kurtz C.B., Millet Y.A., Puurunen M.K., Perreault M., Charbonneau M.R., Isabella V.M., Kotula J.W., Antipov E., Dagon Y., Denney W.S. (2019). An Engineered *E. coli* Nissle Improves Hyperammonemia and Survival in Mice and Shows Dose-Dependent Exposure in Healthy Humans. Sci. Transl. Med..

[37] Sedlmayer F., Hell D., Müller M., Ausländer D., Fussenegger M. (2018). Designer Cells Programming Quorum-Sensing Interference with Microbes. Nat. Commun..

[38] Wu S.-Y., Wen Y., Serre N.B., Laursen C.C.H., Dietz A.G., Taylor B.R., Drobizhev M., Molina R.S., Aggarwal A., Rancic V. (2022). A Sensitive and Specific Genetically-Encoded Potassium Ion Biosensor for in Vivo Applications across the Tree of Life. PLoS Biol..

[39] Velapoldi R.A., Tønnesen H.H. (2004). Corrected Emission Spectra and Quantum Yields for a Series of Fluorescent Compounds in the Visible Spectral Region. J. Fluoresc..

[40] Abdellaoui S., Milton R.D., Quah T., Minteer S.D. (2015). NAD-dependent dehydrogenase bioelectrocatalysis: The ability of a naphthoquinone redox polymer to regenerate NAD. Chem. Commun..

[41] Tanaka R., Ueoka I., Takaki Y., Kataoka K., Saito S. (1983). High molecular weight linear polyethylenimine and poly(N-methylethylenimine). Macromolecules.

