# Supplementary material for: Development of Malate Biosensor-Containing Hydrogels and Living Cell-Based Sensors
Source: Int J Mol Sci. 2024 Oct 16;25(20):11098. doi: 10.3390/ijms252011098 (PMC11507523; doi:10.3390/ijms252011098)
Supplement: Supplementary file 1 [file ijms-25-11098-s001.zip › ijms-3218166-supplementary.pdf]

Materials and Methods

General Transformation Protocol

A 25 µL aliquot of chemically competent frozen BL21* (DE3) or XL1-Blue *E. coli* (Berkeley Macrolab) was combined with 1 µL of plasmid at a concentration of at least 20 ng/µL. For co-transformations with two plasmids, 1 µL of each plasmid was added to the frozen cell aliquot. The aliquot was allowed to thaw on ice for 30 min. The cells were then heat shocked at 42 °C for 45 seconds on a heat block and immediately placed on ice for 2 minutes. Afterward, 300 µL of SOC media were added, and the cells recovered for 45 minutes in an incubator set at 37 °C with shaking at 250 rpm. After recovering, 50 µL of cells and media were plated onto an LB agar plate containing 50 µg/mL of the antibiotic carbenicillin. In the case of co-transformations, the full 300 µL of recovered cells and media mix were plated on LB agar plates containing 50 µg/mL of carbenicillin and spectinomycin, respectively. The plates were allowed to incubate at 37 °C overnight or for 16–18 hours.

General Plasmid Construction Protocol

Two-piece Gibson assembly was used to create plasmids encoding new biosensor constructs, mutants, or citric acid cycle enzymes. The two PCR products were designed to contain overlapping regions that allowed for ligation of both products. Plasmids with single or double amino acid mutations were built by encoding the mutations in the primer overhangs. Plasmids expressing new proteins, such as CitT, fumarase, MDH, and DcuS, were built by using *E. coli* genomic DNA as a template to amplify the gene. Insertion into the desired plasmid was achieved by use of primer overhangs complementary to the plasmid backbone. The primers used can be found in Supplementary Table S1.

PCRs were performed using Phusion DNA polymerase (Berkeley Macrolab) in a 50 µL reaction volume (29 µL sterile water, 10 µL 5x Phusion HF buffer, 2.5 µL each of 10 µM forward and reverse primers, 1 µL template DNA at a concentration of ~10 ng/µL, 5 µL 2.5 mM dNTPs, and 0.5 µL of the polymerase). Thermocycling conditions were 98 °C initial denaturation for 30 s, followed by 34 cycles at 98 °C for 30 s for denaturing, annealing for 30 s (specific annealing temperatures were calculated for each primer pair using the NEB Tm calculator), and extension at 72 °C for 150 s. Thermocycling was completed with a final extension at 72 °C for 5 min.

Agarose gels were used to verify DNA amplification at the correct size using 10 µL of the PCR product. The remaining 40 µL of DNA product was treated with 1 µL of Dpn1 digest enzyme (New England Biolabs) and incubated at 37 °C for 1 h. The DNA was then purified using Qiagen PCR cleanup columns and quantified using a Thermo Scientific Nanodrop 8000.

A 5 µL mixture of the two PCR products was combined with 5 µL of NEB 2x Gibson assembly master mix. Different DNA ratios were used for the Gibson assembly, including 1:2, 2:1, and 1:1 ratios, with a total DNA concentration of 0.02–0.5 pmol. The reaction mixture was incubated in a thermal cycler at 50 °C for 1 h, then transformed into XL1-Blue *E. coli* cells (Berkeley Macrolab). After overnight growth on agar plates containing 50 µg/mL of carbenicillin for pBAD plasmids or 50 µg/mL spectinomycin for pCDF plasmids, single colonies were selected and grown overnight in 3 mL of LB medium supplemented with 50 µg/mL of the appropriate antibiotic. The plasmid was extracted from the overnight culture using a Qiagen miniprep column and the sequence was verified by whole plasmid sequencing (Plasmidsaurus).

Construction and Linker Optimization of DcuS–cpGFP

For the initial construction of DcuS–cpGFP, the cache domain from the DcuS gene was amplified from *E. coli* genomic DNA using NJR3/NJR4 primers containing overlaps with the GFP portion of Citron. The pBAD plasmid backbone containing cpGFP was amplified from the pBAD Citron plasmid (Addgene #134303) using NJR5/NJR6 primers. Two-piece Gibson assembly was performed, and the reaction was transformed into XL1-Blue *E. coli* cells.

To produce a fluorescent and responsive DcuS–cpGFP construct, the two linkers connecting DcuS to cpGFP were optimized sequentially using primers that randomized the two amino acid linker sequence as NNNNNN. The two-piece Gibson assembly protocol described above was repeated with NJR11/NJR4 (linker 1) or NJR3/NJR12 primers (linker 2), and the reaction was transformed into XL1-Blue *E. coli* cells. Each colony represents a different linker sequence.

To rapidly screen each construct with a randomized linker, single colonies were picked, streaked out onto an LB agar plate supplemented with 50 ug/mL carbenicillin, and then grown overnight. The following day, colony streaks were picked and added to 300 μL of LB supplemented with carbenicillin and 1% arabinose and grown overnight at 37 °C 250 rpm in 96 DeepWell plates (Thermo Scientific™ Abgene™ 96 Well 2.2 mL Polypropylene DeepWell™ plates). After overnight growth, the plates were spun down at 4600 rcf for 5 minutes and the cell pellets were resuspended in lysis buffer (100 mM potassium phosphate buffer, pH 7.5, 5% glycerol, 0.4% triton X-100, 2 mM EDTA, 1mg/mL lysozyme). The cells were incubated at 10 °C while shaking at 250 rpm for 1 h to lyse the cells. To a 96-well plate were added 90 μL of cell lysate and 10 μL of PBS buffer with or without malate (100 mM final concentration). The lysate reactions were allowed to equilibrate for 10 min before fluorescence was measured in a SpectraMax i3x plate reader (Molecular Devices) with an ex/em of 485/535 nm. The ΔRFU/RFU was calculated by taking the difference between the average fluorescence of wells with and without malate, then dividing by the average fluorescence of wells without malate.

Linker 1 was randomized first, and ~200 colonies were tested using the cell lysate screen. The colony with the best activation upon malate addition was chosen as the starting template for the randomization of linker 2. For linker 2 randomization, ~200 colonies were again screened.

Malon and Biosensor Mutant Construction

To construct Malon, pBAD Citron was used as a template with five sets of primers designed to introduce mutations in the citrate-binding site (S268A, K253F, S228I, M223F, G204T). Primers NJR13, 15, 17, 19, and 21 were paired with NJR1, and NJR14, 16, 18, and 20 were paired with NJR2. The primer sets were used sequentially after obtaining sequence confirmation of each mutation by whole plasmid sequencing.

An identical protocol as described above was followed to construct mutants of Malon or DcuS–cpGFP. The primers used are described in Supplemental Table S1.

General Protein Purification Protocol

Single colonies of *E. coli* XL1-Blue cells containing the sequence-confirmed construct encoded in the pBAD plasmid were picked and grown overnight in a sterile 12 mL culture tube containing 3 mL LB media supplemented with 50 μg/mL carbenicillin in a 37 °C incubator shaking at 250 rpm. The 3 mL overnight culture was added to a 3 L baffled flask with 1 L of 2x YT media with 50 μg/mL carbenicillin. This large culture was grown until the OD ~0.5-0.8 (typically 3-6 h), then arabinose was added to a final concentration of 0.1%. The induced culture was incubated in a 250 rpm shaker at 20 °C for 16 h. The cells were collected by spinning down at 4700 rcf for 30 min. The spent media was

discarded, and the cell pellet was resuspended in 50 mL of lysis buffer (50 mM sodium phosphate, 300 mM NaCl, 10 mM imidazole, pH 8.0) with the addition of 10 mg lysozyme (Millipore Sigma) and 150 μL of 200 mM PMSF dissolved in ethanol (Sigma Aldrich). The cell lysate was transferred to a 50 mL falcon tube and disrupted for 4 min, with 2 s pulses on and 2 s off with a 70% amplitude using a sonicator (Fisherbrand). The sonicated cells were centrifuged again at 9600 rcf for 40 min at 4 °C to obtain clarified lysate, which was decanted into a new 50 mL falcon tube and incubated with 2 mL of Hispur Nickel NTA resin (Thermo Scientific) for 1 h with rotation at 4 °C. The lysate and resin mixture were added to an Econo-Pac 20 mL chromatography column (Bio Rad) to collect the resin in the column and remove the flow-through. The nickel resin was washed with 60 mL of wash buffer (1x PBS, 25 mM imidazole, pH 8.0) followed by elution of bound protein with 8 mL of elution buffer (250 mM imidazole, pH 8.0). The eluted protein was concentrated and buffer was exchanged with 100 mM potassium phosphate, pH 7.5 with 10% glycerol, using Amicon Ultra Centrifugal Filter columns with a 10 kDa cutoff with repeated centrifugation at 4600 rcf. The concentration of the final protein was estimated using a Bradford assay (Thermo Scientific Pierce BCA Protein Assay Kit) following the kit protocol. Protein purity was analyzed on a Bio-Rad TGX FastCast Acrylamide gel (Figure S1). If the SDS PAGE gel showed impure protein after one Ni-NTA column, protein samples were incubated again with 1 mL of Hispur Nickel NTA resin for 1 h followed by resin washing, elution, buffer exchange, concentration, and analysis on acrylamide gel. After purification, protein samples were stored in aliquots at –80 °C, thawed at room temperature immediately before use, and discarded after 1 freeze–thaw cycle.

Thermoshift Assays

Thermoshift assays were performed on the DcuS cache domain alone. A DcuS pBAD plasmid (containing residues 148–277 of DcuS–cpGFP, only the cache domain) was constructed with a 6x His tag at the N terminus by Gibson assembly using primers NJR7/8 to amplify DcuS from *E. coli* genomic DNA and NJR9/10 to amplify the pBAD backbone. The protein was purified as described above. In transparent 96-well PCR plates (Thermo Scientific AB-0600 non-skirted 96-well PCR plate), 25 μL reactions were prepared. Each well contained 1x SYPRO orange protein gel stain, 1 mM purified DcuS, and 1 mM ligand (malate, fumarate, or citrate) in 100 mM potassium phosphate pH 7.5 buffer. Samples were analyzed using a Bio-Rad CFX Opus 96 Real-Time PCR system. The melt curve was measured from 10.0 °C to 95 °C in increments of 0.5 °C over 10 s using the FRET channel per the manufacturer's instructions.

Docking Analysis

Computational analyses were performed at the Center for High Performance Computing (CHPC) at the University of Utah. Atoms corresponding to malate were manually removed from the PDB file of the DcuS cache (3by8). Using AutoDock Vina and the array of functions available, fumarate was docked into the DcuS cache. The cache PDB file was prepped by using the prepare_recptor4.py function to add hydrogens. Residues within 10 Å of the original malate site were identified and prepared to be treated as flexible amino acids by using the prepare_flexreceptor4.py function. A PDB file containing fumarate was prepared by using the prepare_ligand.py function. Finally, AutoDock Vina was used to dock fumarate into the DcuS-binding pocket that malate had previously occupied. The settings of the Vina run used were an exhaustiveness of 100, the --flex argument, XYZ coordinate centers as the average XYZ coordinates that the atoms from the removed malate ligand occupied, and XYZ search space dimensions of 15 Å.

In Vitro Fluorescence Experiments

All in vitro assays were performed using biosensor proteins purified by the His tag protein purification procedure described above (Figure S1). In vitro assays were performed using 1 μM of biosensor in 100 μL total reaction volume (100 mM potassium phosphate buffer, pH 7.5) in wells of black 96-well plates (Greiner) and analyzed using the

SpectraMax i3x plate reader (Molecular Devices) with ex/em at 485/535 nm. All samples were analyzed in technical triplicate.

ΔRFU/RFU Experiments: Citric acid cycle ligands (malate, fumarate, citrate, etc.) were added to a final concentration of 100 mM to wells containing biosensor protein in buffer. Controls were performed by adding an equal volume of buffer instead of the ligand. The ΔRFU/RFU was calculated by taking the difference between the fluorescence of wells with and without malate, then dividing by the average fluorescence of wells without malate.

K_d Assays: K_d assays were performed by the addition of 10 μL of 10X malate stocks at concentrations ranging from 1 μM to 1 M to 90 μL of a solution containing biosensor protein in buffer. The binding reactions were allowed to equilibrate for 15 min prior to analysis on the plate reader. The K_d was calculated using GraphPad Prism software.

Excitation Emission Spectra: Bound and unbound solutions of Citron, DcuS–cpGFP, and Malon were prepared and measured using the Spectramax i3x plate reader. An excitation scan was performed where the emission wavelength was kept constant at 545 nm, while the excitation wavelengths were varied from 450 nm to 510 nm at intervals of 1 nm, with fluorescence readings taken at each interval. An emission scan was then performed where the excitation wavelength was kept constant at 475 nm, and fluorescence emissions were recorded from 500 nm to 560 nm at 1 nm intervals. Fluorescence values for both the excitation and emission scans were normalized and plotted.

Extinction Coefficient Calculations: A Thermo 8000 UV-vis spectrophotometer was used to measure absorption spectra of purified Citron, Malon, and DcuS–cpGFP. Prior to measurement, all proteins were diluted to 150 μM. Additionally, 5 μL of Malon and DcuS–cpGFP were added to 5 μL of 100 mM malate in potassium phosphate buffer and allowed to incubate for 10 minutes on ice. After incubation on ice with malate, the absorption spectra of both Malon and DcuS–cpGFP were again measured. Absorption, along with protein concentration and the spectrophotometer path length, was used to calculate the molar extinction coefficient of Malon and DcuS–cpGFP in the presence and absence of malate.

Quantum Yield Calculations: The quantum yields of Malon and DcuS–cpGFP were calculated fluorometrically relative to Citron as a reference standard with a known quantum yield [13]. Calculations of quantum yield for the bound and unbound state were carried out as previously described [39]. Briefly, using the Spectramax i3x plate reader, an emission sweep was performed. With a constant excitation wavelength of 485 nM, the fluorescence emissions were recorded from 505 nM to 600 nM at 1 nM intervals for all samples. The emission spectra for each sample were then integrated and the relative quantum yield was calculated using the given equation:

$$Q = Q_r \times \frac{I}{I_r} \times \frac{A_r}{A} \times \frac{n^2}{n_r^2}$$

where Q is the quantum yield, I is the integrated values from of the emission spectra, A is the absorbance at the excitation wavelength, and n is the refractive index of the solution. The subscript r denotes Citron, the reference fluorophore. Absorbance values obtained from calculating the molar extinction coefficient were used.

pH Sensitivity: Malon was diluted into a series of potassium phosphate buffers ranging from pH 4 to 11 and fluorescent measurements were taken using the Spectramax i3x plate reader. Fluorescence vs. pH of Malon with and without malate was plotted.

Kinetic Assays: Using the injector modality of the Spectramax i3x plate reader, the fluorescence of a 90 μL solution containing biosensor protein in buffer was monitored for an initial 30 s at 0.5 s intervals, followed by injection of 10 μL of malate (final concentration 15 mM). Fluorescence was then monitored for 15 min at 0.5 s intervals.

To monitor fumarase reaction kinetics, the fluorescence of a 90 μL solution containing biosensor protein and fumarase (final concentrations 100 nM or 1 mM) in buffer was monitored for an initial 30 s at 0.5 s intervals, followed by injection of 10 μL of fumarate (final concentration 18 mM). Fluorescence was then monitored for 15 min at 0.5 s intervals.

To monitor forward MDH reaction kinetics, the fluorescence of a 90 μL solution containing biosensor protein, MDH (final concentration 1 mM), and malate (final concentration 50 mM) in buffer was monitored for an initial 30 s at 0.5 s intervals, followed by injection of 10 μL of NAD+ (final concentration 50 mM). To monitor reverse MDH reaction kinetics, the fluorescence of a 90 μL solution containing biosensor protein, MDH (final concentration 1 mM), and NADH (final concentration 50 mM) in buffer was monitored for an initial 30 s at 0.5 s intervals, followed by injection of 10 μL of oxaloacetate (final concentration 50 mM). For both cases, fluorescence was then monitored for 15 min at 0.5 s intervals.

Hydrogel Preparation and Fluorescence Microscopy

The naphthoquinone-functionalized linear poly(ethylenimine) redox polymer (1,2-naphthoquinone-4-glycidyl-LPEI, NQ-LPEI) was synthesized using a previously reported procedure [40]. Diisopropylethylamine (DIPEA, 0.643 mL, 3.69 mmol) was dropwise added to glycidol (30.5 mL, 307 mmol) at room temperature. After 10 minutes of stirring, 1,2-naphthoquinone sulfonic acid sodium salt (800.8 mg, 3.07 mmol) was added slowly to the stirred mixture for a further 10 minutes. For removal of the unreacted reagents, several extractions were conducted first with dichloromethane against a saturated brine solution, and once the organic phase appeared as colorless, all organic fractions were combined and additional extractions were performed with chloroform against water, then dried over magnesium sulfate and finally purified over silica gel with a diethyl ether: dichloromethane gradient. An epoxide-functionalized naphthoquinone derivative was obtained with an approximative yield of 13.1 %.

LPEI (21.5 mg) was synthesized as previously reported and mixed with the obtained epoxide–naphthoquinone derivative (28.7 mg) in stirred methanol (~15 mL) overnight at room temperature [41]. Removal of the solvent was conducted under reduced pressure, and the resulting NQ-LPEI polymer (34.9 mg) was washed with ethyl acetate to remove any residual naphthoquinone.

To prepare electrodes for confocal fluorescence microscopy, stock solutions of 10 mg/mL –1 NQ-LPEI and 13% v/v ethyleneglycol diglycidyl ether (EDGDE) were prepared in pure water. The crosslinked hydrogel containing the Malon sensor was prepared by gently mixing 70 μL of NQ-LPEI, 30 μL of 300 μM Malon sensor, and 4.5 μL of EDGDE. Indium tin oxide (ITO) electrodes were cut into 1 cm x 0.5 cm stripes and used as a platform for the crosslinked hydrogel. A total volume of 20 μL of the polymeric mixture containing the entrapped fluorescent sensor was drop casted on one side of the ITO electrode, in a delimited area of 0.25 cm^2 in two consecutive depositions of 10 μL. After each deposition, the polymer was left to completely dry at 4 °C in the dark to allow the completion of the crosslinking process and the stabilization of the hydrogel before performing further experiments.

Sensor-coated electrodes were placed in a single well of a 96-well plate containing 90 μL of 100 mM potassium phosphate buffer, pH 7.5. Images of the sensor-coated electrodes were taken using a Zeis 880 Airy Scan confocal microscope with a 5x objective. A 488 nm laser was used, and fluorescence spectra were captured using the GFP channel. Both brightfield and fluorescence images were taken. Following initial imaging of the electrode, 10 μL of 500 mM malate were added to the well, to a final malate concentration of 50 mM. The plate was imaged again under the microscope with identical conditions. Brightfield and fluorescent images were processed and cropped by ImageJ. The measurement function was used to quantify fluorescence at marked locations of the hydrogel.

Flow Cytometry Analysis of Bacterial Cells Expressing Malon

A CitT pCDF plasmid was constructed by Gibson assembly using primers NJR27/28 to amplify CitT from *E. coli* genomic DNA and NJR41/42 to amplify the pCDF backbone. Malon pBAD and the CitT pCDF plasmids were co-transformed into Bl21* DE3 cells using the transformation conditions described above, and grown overnight on LB agar plates supplemented with 50 μg/mL carbenicillin and 50 μg/mL spectinomycin. Twelve single colonies were picked and each was resuspended in 3 mL of LB media supplemented with 50 μg/mL carbenicillin, 50 μg/mL spectinomycin, and 1% arabinose in 12 mL culture tubes. IPTG was added to culture tubes to a final concentration of 0, 100 mM, 1 mM, or 2 mM (three biological replicates for each condition). The cultures were induced at 37 °C, 250 rpm for 16 h, then gently spun down (2000 g for 5 min) and washed with 3 mL of M9 minimal media, and then resuspended in 3 mL of M9 minimal media. Each culture was split into two 1.5 mL samples, and 150 μL of 1x PBS or malate in 1x PBS (20 mM final concentration) were added to each culture tube. The cultures were incubated with or without malate for 5-45 min; then, to prepare cell samples for flow cytometry analysis, 1 μL of each culture was resuspended in 99 μL of 1x PBS in a 96-well plate.

Cellular fluorescence of up to 30,000 cells was measured using the Attune NXt flow cytometer (Life Technologies) with the 488 nm excitation laser and GFP emission filter. Using FlowJo software, forward and side scatter gating was used to select normal bacterial cells, and the mean fluorescence intensity of the cell population was determined. ΔMFI/MFI was calculated by taking the difference between the MFI values of cell populations with and without malate, then dividing by the MFI value of cell populations without malate. Three biological replicates were measured for each experimental condition.

A

MASKGEELFTGVVPILAELDGDVNGHKFSVSGEGEGDATYGKLTMKFICTTGKLPVPWPTLVTTLTYGVQCFSRY

PDHMRQHDFFKSAMPEGYIQERTIFFKGDGNYKTRAEVKFEGDTLVNRIELKGADFREDGNILGHKLVYNSAISD

MTRDGLANKALAVARTLADSPEIRQGLQKKPQESGIQAIAEAVRKRNDLLFIVVTDMQSLRYSHPEAQRIGQPFK

GDDILKALNGEENVAINRGFLAQALRVFTPIYDENHKQIGVVAIGLELSRVTSYLVYIKADKQKNGIKANFHVRH

NIEDGGVQLAYHYQQNTPIGDGPVLLPDNHYLSVQSELSKDPNEKRDHMVLQEHVTAAGITLGMAELFK

B

MASKGEELFTGVVPILAELDGDVNGHKFSVSGEGEGDATYGKLTMKFICTTGKLPVPWPTLVTTLTYGVQCFSRY

PDHMRQHDFFKSAMPEGYIQERTIFFKGDGNYKTRAEVKFEGDTLVNRIELKGADFREDGNILGHKLVYNMVTEE

RLHHQVGQRALIQAMQISTMPELVEAVQKRDLARIKALIDPMRSFPDATYITVGDASGQRLYHVNPDEIGKSMVG

GDSDEALINAKGYVSVRKGSLGSSLRGKSPILDATGRVVGIVSVGYTIEQLESNYIKADKQKNGIKANFHVRHNI

EDGGVQLAYHYQQNTPIGDGPVLLPDNHYLSVQSELSKDPNEKRDHMVLQEHVTAAGITLGMAELFK

C

MASKGEELFTGVVPILAELDGDVNGHKFSVSGEGEGDATYGKLTMKFICTTGKLPVPWPTLVTTLTYGVQCFSRY

PDHMRQHDFFKSAMPEGYIQERTIFFKGDGNYKTRAEVKFEGDTLVNRIELKGADFREDGNILGHKLVYNMVTEE

RLHHQVGQRALIQAMQISTMPELVEAVQKRDLARIKALIDPMRSFPDATYITVTDASGQRLYHVNPDEIGKSFVG

GDIDEALINAKGYVSVRKGSLGSSLRGFSPILDATGRVVGIVAVGYTIEQLESNYIKADKQKNGIKANFHVRHNI

EDGGVQLAYHYQQNTPIGDGPVLLPDNHYLSVQSELSKDPNEKRDHMVLQEHVTAAGITLGMAELFK

D

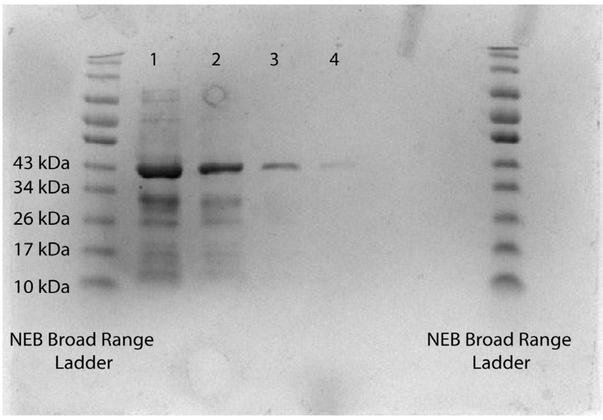

Supplementary Figure S1: Sequences of (A) DcuS–cpGFP, (B) Citron, and (C) Malon. GFP sequences are in green. Cache domain sequences are in black. Linker regions are in blue. Mutations to convert Citron to Malon are in red. (D) SDS PAGE gel of purified biosensor proteins with the NEB Broad Range Ladder. Lanes 1 and 3 are Malon (45 kDa) after one or two Ni-NTA column purifications, respectively. Lanes 2 and 4 are DcuS–cpGFP (45 kDa) after one or two Ni-NTA column purifications.

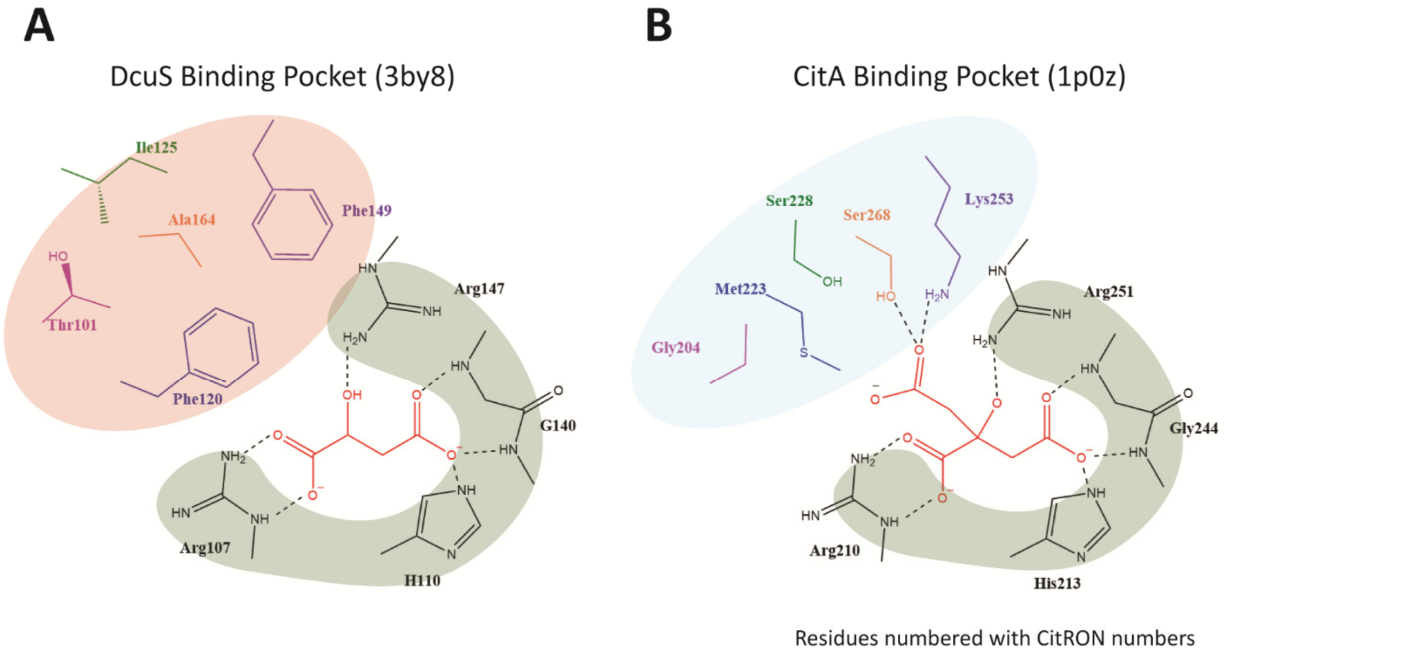

Supplementary Figure S2: Comparison of the DcuS-binding pocket (A) to the CitA-binding pocket (B). Aside from structural homology, both pockets share a high degree of sequence homology, with both containing core residues to bind citrate and malate (highlighted in green). The two proteins differ in that DcuS contains more bulky non-polar residues (highlighted in red) and CitA contains less bulky polar residues (blue) that make polar contact with the carboxylic acid moiety present on citrate. Mutation of these five polar residues in the Citron pocket to the homologous non-polar residues found in DcuS yielded Malon.

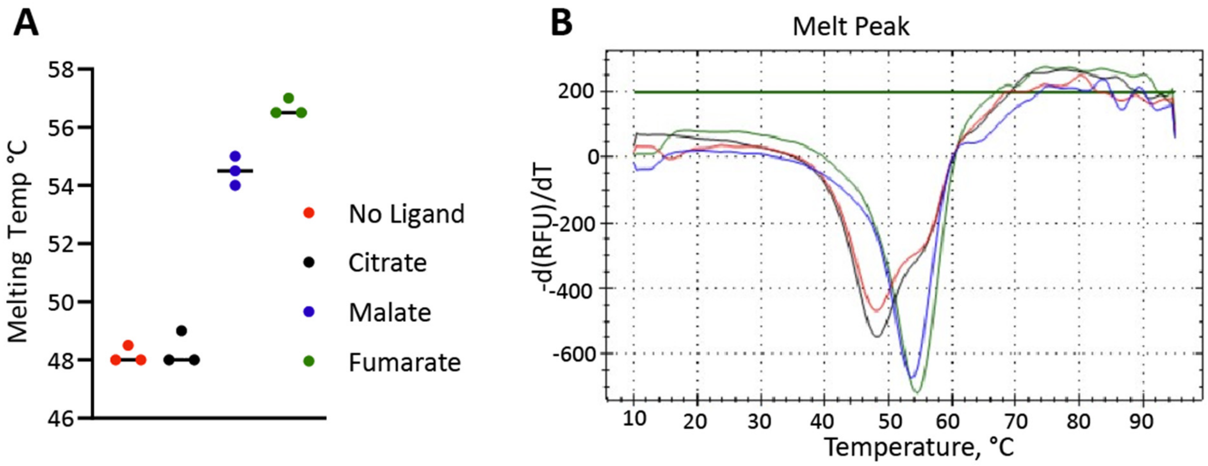

Supplementary Figure S3: Thermal shift data for DcuS. Shown are data for 3 technical replicates.

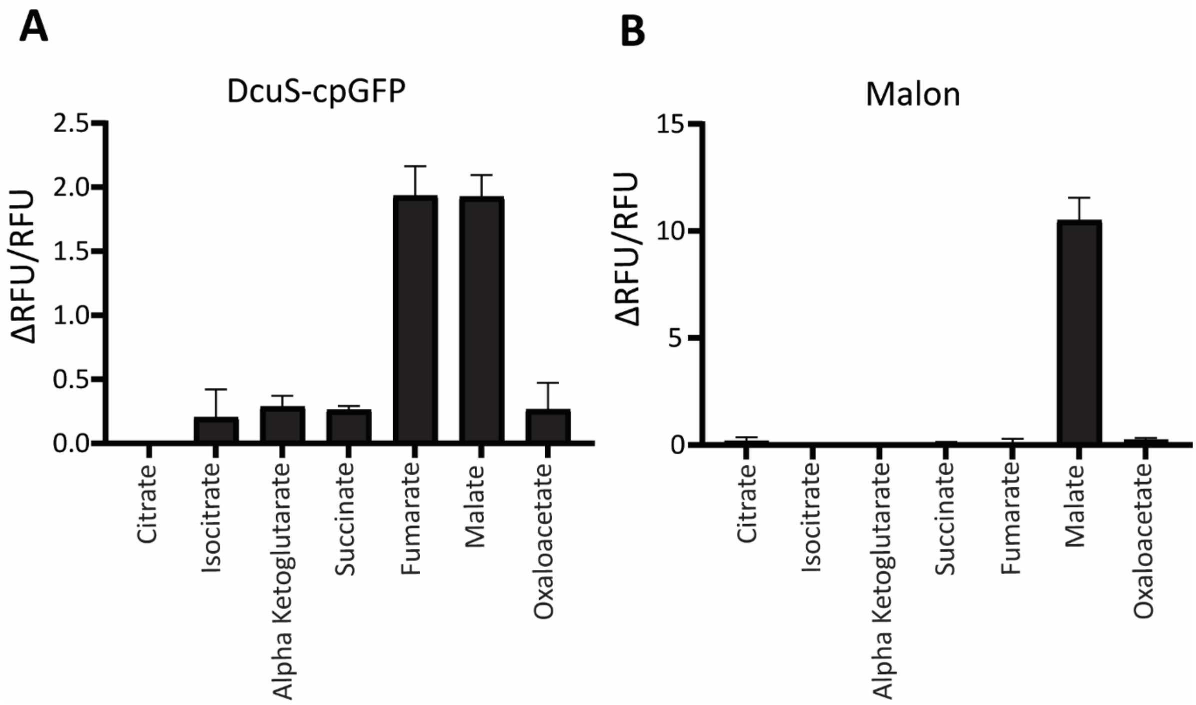

Supplementary Figure S4: ΔRFU/RFU for (A) DcuS–cpGFP and (B) Malon in response to 100 mM of malate and other Krebs cycle intermediates. Shown are data for 3 technical replicates with standard deviation.

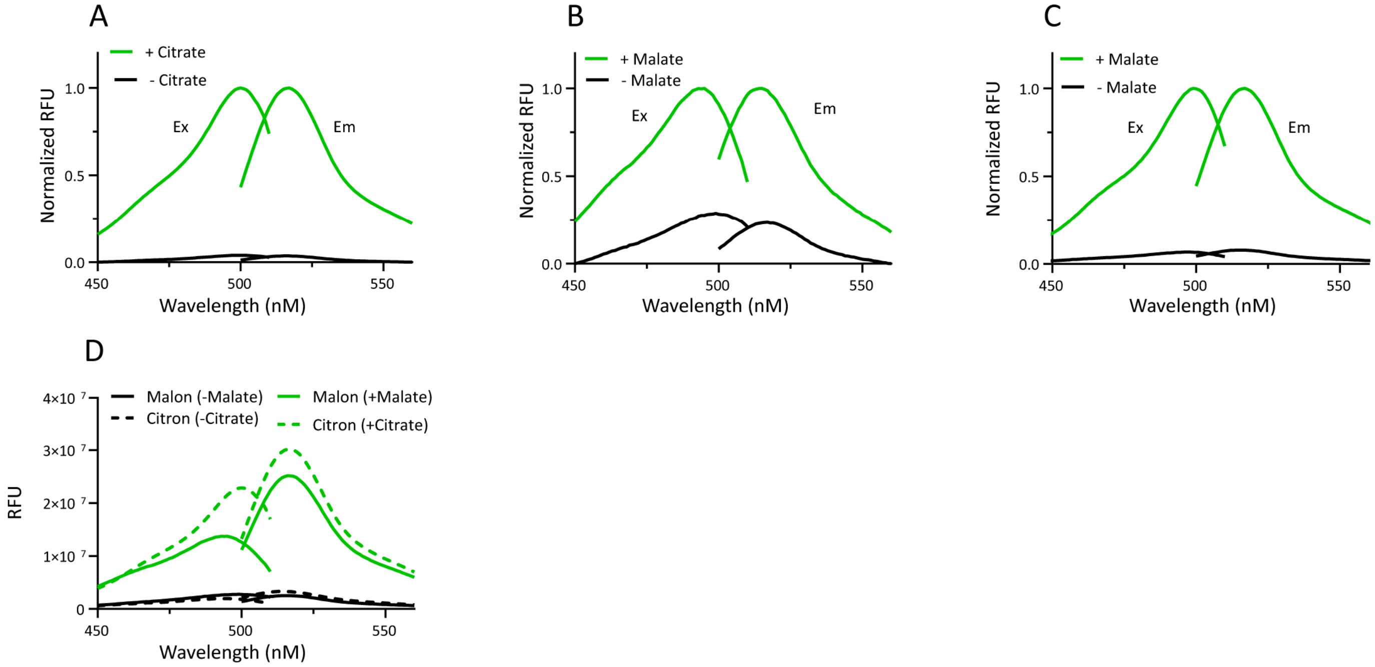

Supplementary Figure S5: Normalized excitation/emission profiles in the presence and absence of ligand for (A) Citron, (B) DcuS–cpGFP, (C) Malon, and (D) the raw excitation/emission profiles for Malon and Citron in the presence and absence of malate or citrate.

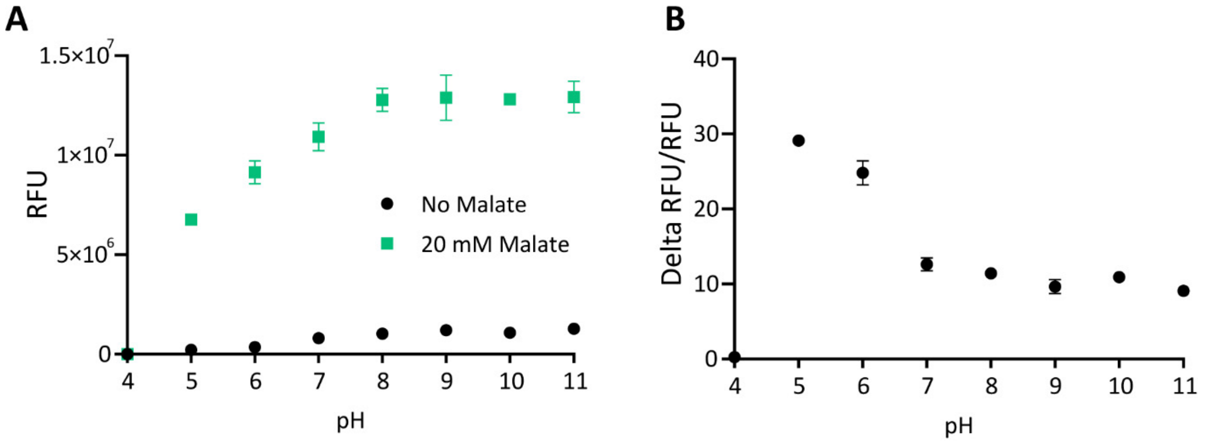

Supplementary Figure S6: pH dependence of the Malon biosensor. (A) Raw fluorescence of the Malon sensor with and without malate between pH 4 and 11; (B) delta RFU/RFU profile of the Malon sensor at various pHs.

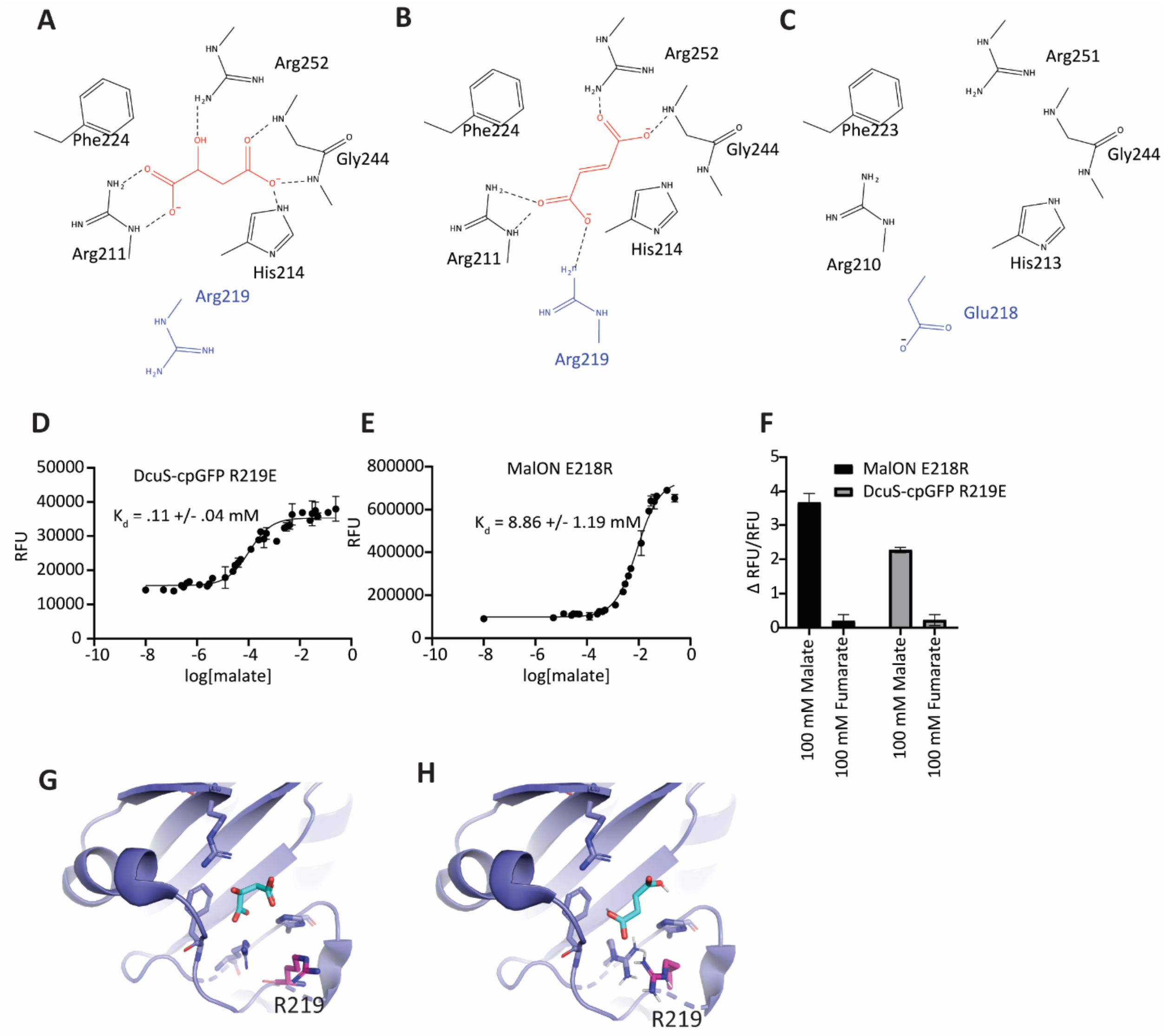

Supplementary Figure S7: Assessment of substrate specificity between DcuS–cpGFP and Malon.
(A) Schematic of binding pocket of DcuS with bound malate (PDB 3by8) (residue numbers are numbering the DcuS–cpGFP construct). (B) Malate was removed from the binding pocket, and fumarate was docked into the pocket. When near residues were allowed to relax to the most stable position, Arg219 repositioned to make contact with fumarate. (C) By homology, the binding pocket of Malon contains Glu218 in the same position as Arg219. (D, E) Binding affinity to malate for the R219E mutant for DcuS–cpGFP and the E218R mutant for Malon. (F) Selectivity of these mutant biosensors for malate and fumarate. (G, H) Structures of malate and fumarate (modeled) bound to WT DcuS related to parts A and B. Fumarate and malate shown in cyan.

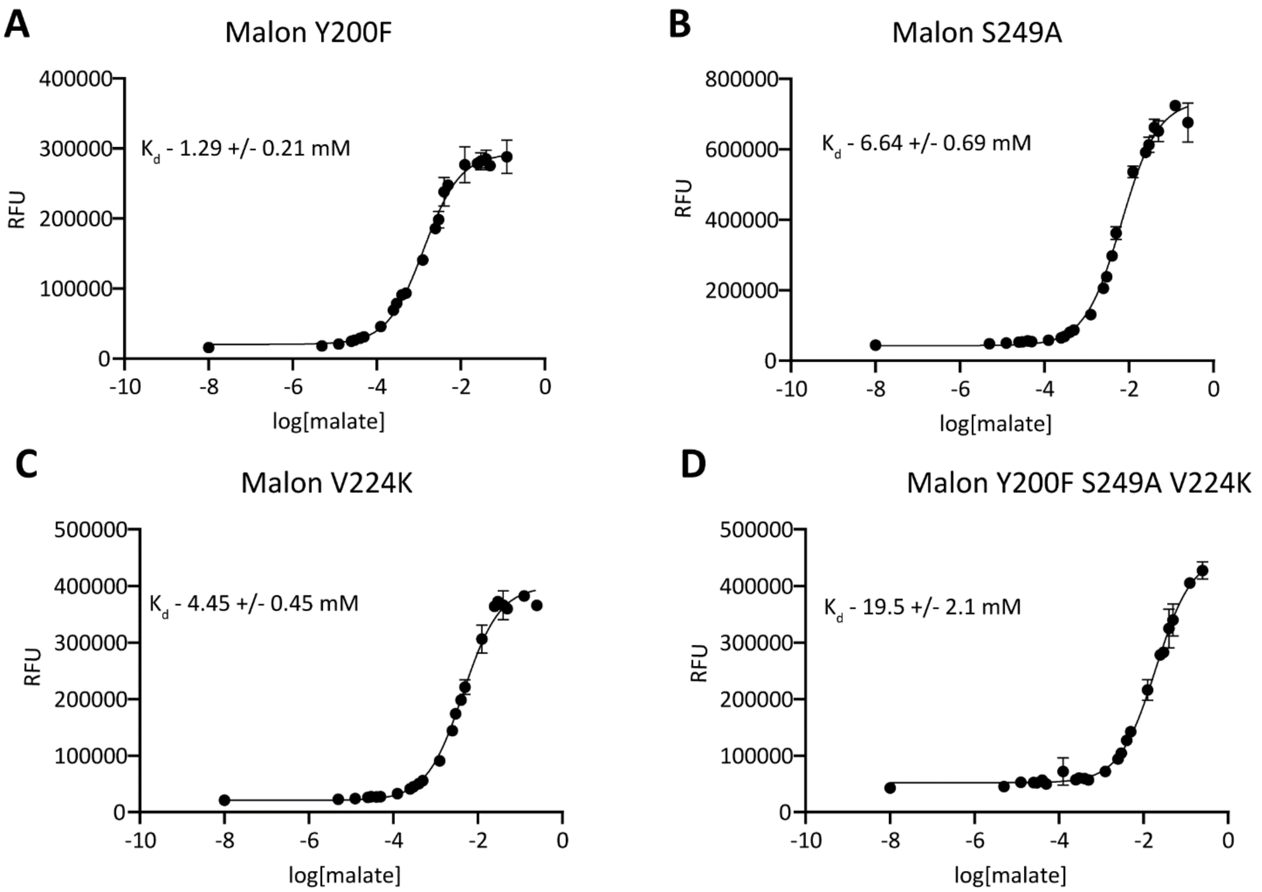

Supplementary Figure S8: Mutations made in the secondary coordination sphere of Malon to make the binding pocket more similar to the DcuS pocket. Four constructs were tested: (A) Y200F, (B) S249A (C) V224K, and (D) all 3 mutations combined. Shown are data for 3 technical replicates with standard deviation.

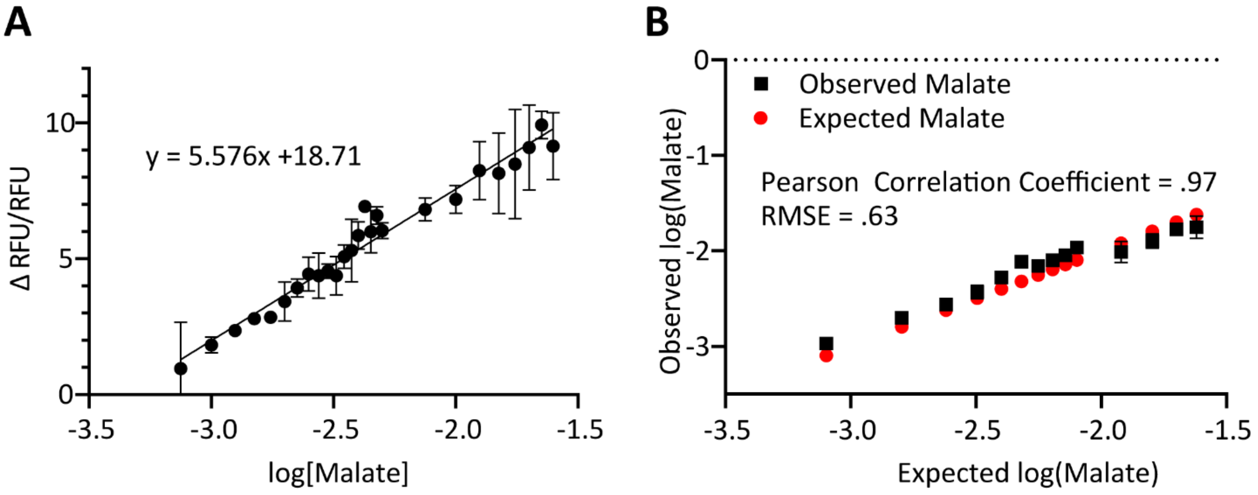

Supplementary Figure S9: (A) Regression curve for Malon response to malate. (B) Malon was used to measure the fluorescence of a series of enzyme reactions with known concentrations of fumarase and fumarate to produce malate. The observed malate concentration was calculated using the regression curve shown in part A (black points) and compared to the expected malate values determined using the known equilibrium constant for the fumarase reaction (4.4) *[28]*.

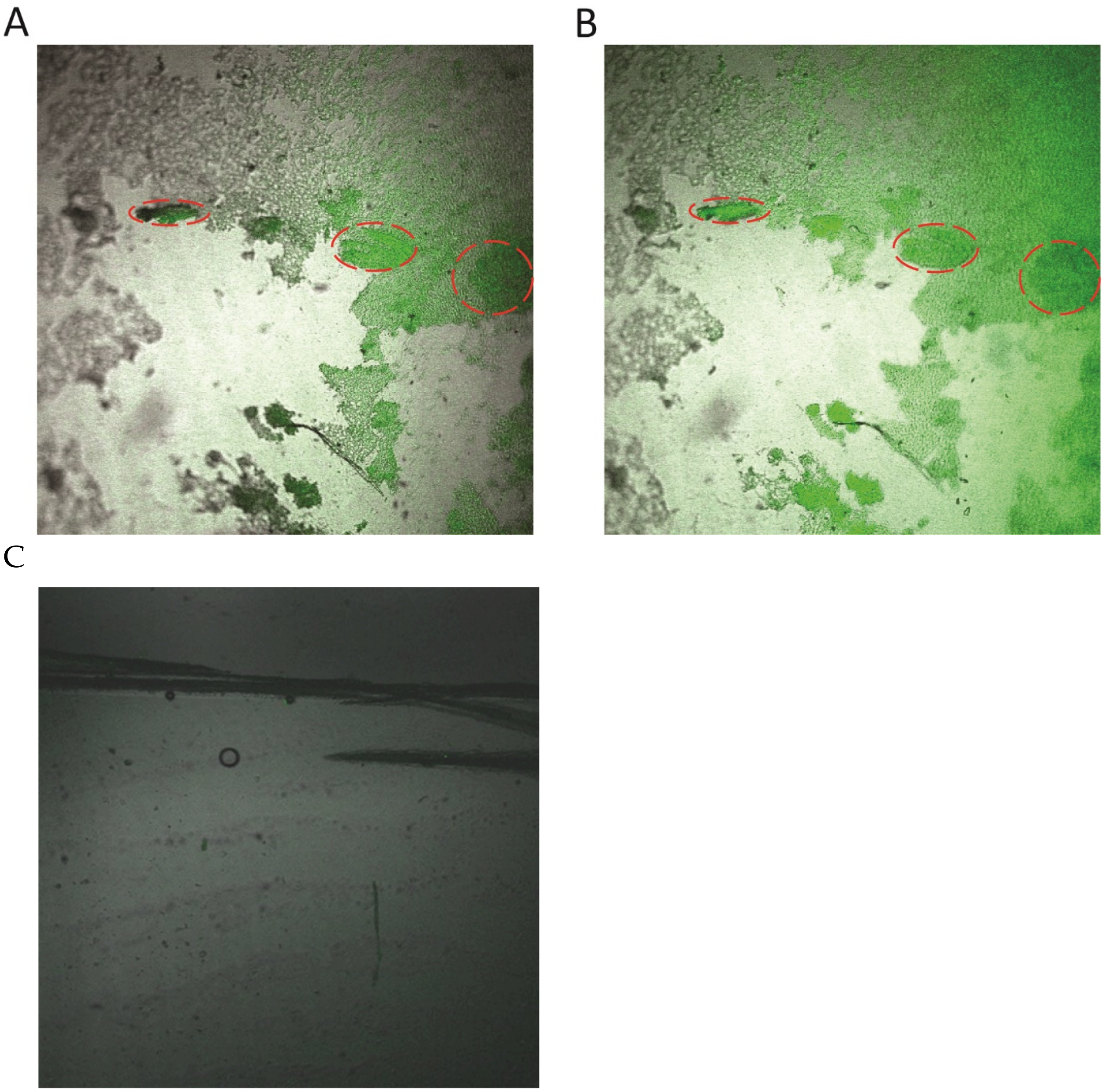

Supplementary Figure S10: Full image of Malon-containing hydrogel (A, B) and control hydrogel without Malon (C) cast on an electrode obtained from confocal fluorescence microscopy. Areas circled in red were used to quantify the fluorescence of hydrogel +/- malate. (A) Electrode in buffer. (B) Electrode in buffer + 50 mM malate. (C) Electrode in buffer.

Table S1. Primers used in this study.

Number	Sequence	Description	Gibson Information
1	CAGTGCTGCAATGATACCGC	pBAD backbone amplification primer	
2	AGACTGGATGGAGGCGGATA	pBAD backbone amplification primer	
3	GCACAAGCTGGTGTACAACATGGTGATTAGCGATATGACCCGTGATGG	DcuS amplification, pairs with primer 3	Combined with product of 5–6 for Gibson
4	CTTGATATAGACCAAATTCGAGGTAACACGGCTCAGTTCCA	DcuS amplification, pairs with primer 4	
5	TTGGTCTATATCAAGGCCGACAAA	Citron backbone amplification, pairs with primer 6	Combined with product of 3–4 for Gibson
6	GTACACCAGCTTGTGCCCCA	Citron backbone amplification, pairs with primer 5	
7	TGACGATAAGGATCCGAGCTCGAGAATCAGTGATATGACGCGAGATGG	DcuS amplification, pairs with primer 8	Combined with product of 9–10 for Gibson
8	ATCCGCCAAAACAGCCAAGCTTTTACCAGCGACTGTCATTGATCT	DcuS amplification, pairs with primer 7	
9	TAAAAGCTTGGCTGTTTTGGCG	pBAD backbone amplification, pairs with primer 10	Combined with product of 7–8 for Gibson
10	TCTCGAGCTCGGATCCTTATC	pBAD backbone amplification, pairs with primer 9	
11	GCACAAGCTGGTGTACAACNNNNNNATTAGCGATATGACCCGTGATGG	Randomization of DcuS	Combined with product of 5–6 for Gibson
12	CTTGATATAGACCAANNNNNNGGTAACACGGCTCAGTTCCA	Randomization of DcuS	
13	CATTGTGGCGGTAGGCTATACCATTGAGCAACTGGAATCG	Citron to Malon mutation S268A, pairs with primer 1	Combined with product of 2–14 for Gibson
14	CAATGGTATAGCCTACCGCCACAATGCCGACCACCCTG	Citron to Malon mutation S268A, pairs with primer 2	Combined with product of 1–13 for Gibson
15	CGTCGCTGCGCGGTTTTTCGCCGATTCTGGACGCAACCG	Citron to Malon mutation K253F, pairs with primer 1	Combined with product of 2–16 for Gibson
16	AATCGGCGAAAAACCGCGCAGCGACGATCCCAGCGAG	Citron to Malon mutation K253F, pairs with primer 2	Combined with product of 1–15 for Gibson
17	CGGCGATATCGATGAGGCGTTGATAAACGCTAAAGGCTACGTG	Citron to Malon mutation S228I, pairs with primer 1	Combined with product of 2–18 for Gibson
18	TCAACGCCTCATCGATATCGCCGCCTACAAACGATTTGCC	Citron to Malon mutation S228I, pairs with primer 2	Combined with product of 1–17 for Gibson
19	TGAAATCGGCAAATCGTTTGTAGGCGGCGATATCGATGAG	Citron to Malon mutation M223F, pairs with primer 1	Combined with product of 2–20 for Gibson

20	TCGATATCGCCGCCTACAAACGATTTGCCGATTTCATCAGGA	Citron to Malon mutation M223F, pairs with primer 2	Combined with product of 1–19 for Gibson
21	TCACCGTCACTGATGCCAGCGGCCAGCGCCTCTATCAC	Citron to Malon mutation G204T, pairs with primer 1	Combined with product of 2–22 for Gibson
22	GCCGCTGGCATCAGTGACGGTGATGTAGGTGGCGTCGG	Citron to Malon mutation G204T, pairs with primer 2	Combined with product of 1–21 for Gibson
23	GCCGTTTGAAGGTGATGATATTCTGAAAGCACTGAATGGT	DcuS–cpGFP mutation K225E, pairs with primer 1	Combined with product of 2–24 for Gibson
24	TTCAGAATATCATCACCTTCAAACGGCTGACCAATACGCT	DcuS–cpGFP mutation K225E, pairs with primer 2	Combined with product of 1–23 for Gibson
25	AAGCACAGCGTATTGGTAAGCCGTTTGAAGGTGATGATATTCTGA	DcuS–cpGFP mutation Q222K, pairs with primer 1	Combined with product of 2–26 for Gibson
26	ACCTTCAAACGGCTTACCAATACGCTGTGCTTCCG	DcuS–cpGFP mutation Q222K, pairs with primer 2	Combined with product of 1–25 for Gibson
27	AAGTATAAGAAGGAGATATACATATGTCTTTAGCAAAAGA-TAATATATGGAAACTATTGGCCCCAC	CitT amplification A, pairs with primer 28	Combined with product of 41–42 for Gibson
28	GCGGTTTCTTTACCAGACTCGAGTTAGTTCCACATGGCGAGAATCG	CitT amplification A, pairs with primer 27	
29	TTGAGTTTAAACGGTCTCCAGC	Amplification of pBAD backbone, pairs with primer 30	Combined with product of 31–32 for Gibson
30	TGGATCCATATCTCCTTCTTAAAGT	Amplification of pBAD backbone, pairs with primer 29	
31	TAAGAAGGAGATATGGATCCATGAATACAGTACGCAGCGAAA	Fumarase C amplification, pairs with primer 32	Combined with product of 29–30 for Gibson
32	GACCGTTTAAACTCAATGGTGATGGTGATGATGACGCCCGGCTTTCAT-ACTG	Fumarase C amplification, pairs with primer 31	
33	TAAAAGCTTGGCTGTTTTGGCG	Amplification of pBAD backbone, pairs with primer 34	Combined with product of 35–36 for Gibson
34	TCTCGAGCTCGGATCCTTATC	Amplification of pBAD backbone, pairs with primer 33	
35	TGACGATAAGGATCCGAGCTCGAGAATGAAAGTCGCAGTCCTCGG	Malate dehydrogenase amplification, pairs with primer 36	Combined with product of 33–34 for Gibson

36	TCATCCGCCAAAACAGCCAAGCTTTTACTTATTAACGAACTCTTCGCCCA	Malate dehydrogenase amplification, pairs with primer 35	
37	ATCACGTCAATCCTGATCGCATCGGCAAATCGTTTGAAGGC	Malon E218R mutation, pairs with primer 1	Combined with product of 2–38 for Gibson
38	TCAAACGATTTGCCGATGCGATCAGGATTGACGTGATAGAGGC	Malon E218R mutation, pairs with primer 2	Combined with product of 1–37 for Gibson
39	AGCACAGGAAATTGGTCAGCCGTTTAAAGGTGATGATA	DcuS–cpGFP R219E mutation, pairs with primer 1	Combined with product of 2–40 for Gibson
40	CTGACCAATTTCCTGTGCTTCCGGATGGCTATAACG	DcuS–cpGFP R219E mutation, pairs with primer 1	Combined with product of 1–39 for Gibson
41	TCGAGTCTGGTAAAGAAACCG	pCDF backbone amplification, pairs with primer 42	Combined with product of 27–28 for Gibson
42	CTTATACTTAACTAATATACTAAGATGGGGA	pCDF backbone amplification, pairs with primer 41	

References

13. Zhao, Y.; Shen, Y.; Wen, Y.; Campbell, R.E. High-Performance Intensiometric Direct- and Inverse-Response Genetically Encoded Biosensors for Citrate. *ACS Cent. Sci.* **2020**, *6*, 1441–1450.
28. Krebs, H.A. The Equilibrium Constants of the Fumarase and Aconitase Systems. *Biochem. J.* **1953**, *54*, 78–8
39. Velapoldi, R.A.; Tønnesen, H.H. Corrected Emission Spectra and Quantum Yields for a Series of Fluorescent Compounds in the Visible Spectral Region. *J. Fluoresc.* **2004**, *14*, 465–472, https://doi.org/10.1023/b:jofl.0000031828.96368.c1.
40. Abdellaoui, S.; Milton, R.D.; Quah, T.; Minteer, S.D. NAD-dependent dehydrogenase bioelectrocatalysis: the ability of a naphthoquinone redox polymer to regenerate NAD. *Chem. Commun.* **2015**, *52*, 1147–1150, https://doi.org/10.1039/c5cc09161f.
41. Tanaka, R.; Ueoka, I.; Takaki, Y.; Kataoka, K.; Saito, S. High molecular weight linear polyethylenimine and poly(N-methylethylenimine). *Macromolecules* **1983**, *16*, 849–853, https://doi.org/10.1021/ma00240a003.
